# A Deep Learning Radiomics Nomogram to Predict Response to Neoadjuvant Chemotherapy for Locally Advanced Cervical Cancer: A Two-Center Study

**DOI:** 10.3390/diagnostics13061073

**Published:** 2023-03-11

**Authors:** Yajiao Zhang, Chao Wu, Zhibo Xiao, Furong Lv, Yanbing Liu

**Affiliations:** 1 College of Medical Informatics, Chongqing Medical University, Chongqing 400016, China; 18502310801@163.com; 2 Department of Radiology, The First Affiliated Hospital of Chongqing Medical University, Chongqing 400016, China

**Keywords:** deep learning, radiomics nomogram, locally advanced cervical cancer, neoadjuvant chemotherapy

## Abstract

**Purpose:** This study aimed to establish a deep learning radiomics nomogram (DLRN) based on multiparametric MR images for predicting the response to neoadjuvant chemotherapy (NACT) in patients with locally advanced cervical cancer (LACC). **Methods:** Patients with LACC (FIGO stage IB-IIIB) who underwent preoperative NACT were enrolled from center 1 (220 cases) and center 2 (independent external validation dataset, 65 cases). Handcrafted and deep learning-based radiomics features were extracted from T2WI, DWI and contrast-enhanced (CE)-T1WI, and radiomics signatures were built based on the optimal features. Two types of radiomics signatures and clinical features were integrated into the DLRN for prediction. The AUC, calibration curve and decision curve analysis (DCA) were employed to illustrate the performance of these models and their clinical utility. In addition, disease-free survival (DFS) was assessed by Kaplan–Meier survival curves based on the DLRN. **Results:** The DLRN showed favorable predictive values in differentiating responders from nonresponders to NACT with AUCs of 0.963, 0.940 and 0.910 in the three datasets, with good calibration (all *p* > 0.05). Furthermore, the DLRN performed better than the clinical model and handcrafted radiomics signature in all datasets (all *p* < 0.05) and slightly higher than the DL-based radiomics signature in the internal validation dataset (*p* = 0.251). DCA indicated that the DLRN has potential in clinical applications. Furthermore, the DLRN was strongly correlated with the DFS of LACC patients (HR = 0.223; *p* = 0.004). **Conclusion:** The DLRN performed well in preoperatively predicting the therapeutic response in LACC and could provide valuable information for individualized treatment.

## 1. Introduction

Cervical cancer is the most frequently diagnosed cancer among women, with over 500,000 new diagnoses and 300,000 deaths per year [1,2]. In the past, for the treatment of locally advanced cervical cancer (LACC), radical hysterectomy (RH) and chemotherapy were suggested; however, the likelihood of treatment failure is high [3,4]. Since the 1990s, neoadjuvant chemotherapy (NACT), a short-course chemotherapy administered before surgery, has been used as a substitute treatment option for cervical cancer [5]. Although the five-year overall and disease-specific survival rates are similar between these treatment modalities, they demonstrate disparate toxicity profiles [6]. An increasing number of findings have suggested that NACT followed by radical hysterectomy or radiotherapy can improve survival. A meta-analysis from 21 randomized studies found that NACT substantially minimized the risk of death in LACC participants [7], while another analysis of 6 randomized controlled clinical trials demonstrated that NACT significantly improved both overall survival and progression-free survival [8]. The potential advantages of NACT have been reinforced by its ability to reduce tumor volume, eradicate subclinical distant metastasis, allow better delivery of radiation and improve prognosis [9,10]. Current research should focus on how to best identify patients who will benefit the most from this method. Ideally, radiotherapy should be avoided to the greatest extent in young women to preserve ovarian and sexual function. Only individuals with excellent reaction and negative lymph nodes should be subjected to radical surgery. This will decrease the requirement for postoperative chemoradiation and the increased morbidity associated with this approach when combined with radical surgery.

MRI has wide utility in pelvic evaluation due to its unique benefits in soft tissue imaging; it is effective in detecting primary tumor size, local diffusion and lymph node metastasis and is considered the gold standard for monitoring the response of cervical tumors to chemotherapy and determining eligibility for eventual excision [11]. In recent years, MRI-based radiomics has received increasing attention due to the advantages of extracting a large number of quantitative features from imaging phenotypes and offering meaningful biologic information [12,13]. Recent efforts have highlighted that MRI-based radiomics features may hold potential in the prediction of the response to NACT in LACC preoperatively [14,15], confirming that radiomics can distinguish patients who are suitable for NACT from those who are not. While the performance of these radiomics models for the prediction of NACT response is within acceptable ranges, there are still some limitations. Handcrafted radiomics features are restricted to the current knowledge of medical imaging [16] and operators’ prior knowledge and hence may be insufficiently representative. Because generic features might lead to poor classification model performance, most tasks typically require distinguishing features depending on individual context to produce better outcomes. However, such distinguishing characteristics may not be applicable to other activities [17].

Deep learning (DL) performs well in medical image classification and prediction, extracting and learning deep features directly in a data-driven manner [18,19,20], and has shown excellent performance in precision cancer diagnosis, therapeutic decision making and survival analysis [21,22,23]. Since DL provides more abstract and difficult-to-mine radiomics features, several researchers have combined handcrafted and DL-based radiomics features to develop prediction models in cancers [24,25,26]. Although radiomics is capable of quantifying interpretable tumor image features, DL-based features primarily focus on effective semantic information, making them suitable for large-scale image analysis [27,28]. Moreover, preoperative clinical parameters offer additional useful data for predicting the NACT response [29]. Combining clinical characteristics with the two sets of radiomics features can predict NACT effectiveness from multiple perspectives, providing a more comprehensive assessment.

Nevertheless, investigations on the development of a DL radiomic model for predicting the therapeutic response to NACT in patients with LACC are still lacking. Notably, the pathological response was an independent prognostic variable for disease-free survival (DFS) and overall survival in patients with stage Ib2-IIb disease who underwent radical hysterectomy combined with NACT. Patients who are nonresponsive to NACT have a higher risk of recurrence than those who do respond [30]. However, the application of deep learning radiomics to predict the pathological response to NACT has been infrequently described in LACC. In addition, it is essential to determine radiomics and clinical predictors of the NACT response to stratify cervical cancer patients based on relevant therapy.

In this study, we first built and validated a new preoperative deep learning radiomics nomogram (DLRN) that combines handcrafted and DL-based radiomics features and clinical predictors for the prediction of the clinical response to NACT in cervical cancers.

## 2. Materials and Methods

### 2.1. Patients

**Figure 1:**
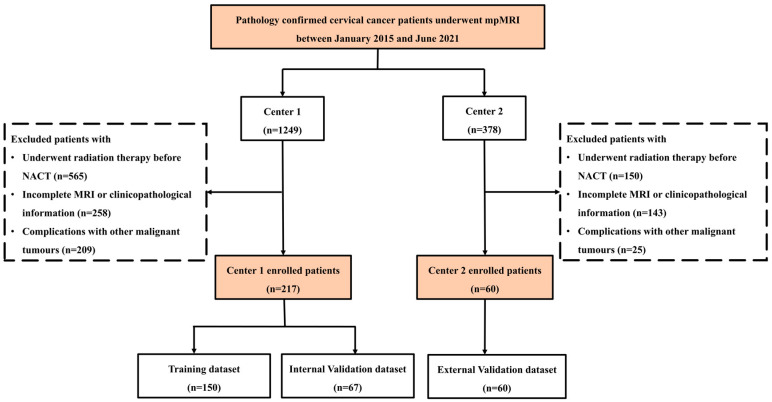
Flowchart of locally advanced cervical cancer (LACC) patients for inclusion in the developmental dataset and three external test datasets.

**Figure 2:**
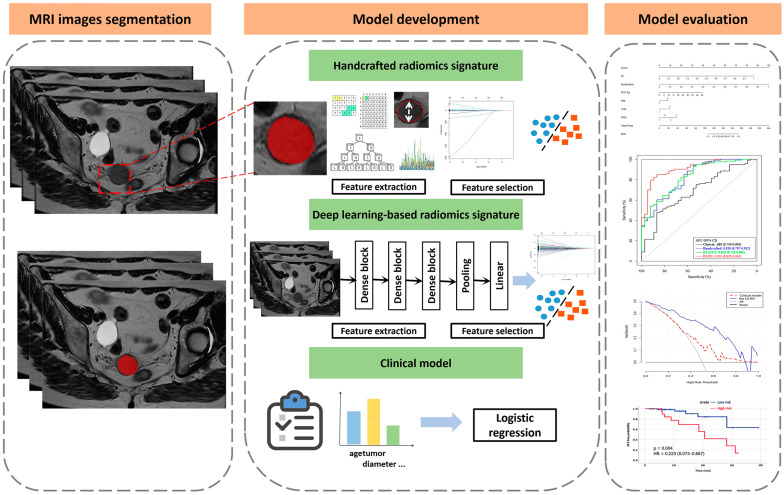
Workflow of this study, including step 1 MR segmentation; step 2 model development: (a) handcrafted radiomics signature, (b) deep learning-based radiomics signature, (c) clinical model; step 3 model evaluation. Receiver operating characteristic (ROC), area under the curve (AUC), calibration, and decision curve analysis (DCA) was used to confirm the performance and clinical utility of model.

A retrospective study was performed after receiving approval from the Ethics Committee of our institution, who waived the need for informed consent from the participants. The study was conducted in accordance with the Declaration of Helsinki. All procedures performed during this study complied with the 1964 Helsinki declaration. The data of patients with cervical cancer with a confirmed histopathology from two centers between January 2015 and June 2021 were enrolled (Figure 1 and Supplementary S1). The inclusion criteria were as follows: (1) conventional pelvic MR scanning examinations performed before NACT, including T2-weighted imaging (T2WI), diffusion-weighted imaging (DWI) and contrast-enhanced (CE)-T1WI, in patients with pathologically confirmed cervical cancer; (2) FIGO stage (2018) from IB to IIIB; (3) use of the same NACT therapy; and (4) the presence of clinicopathological data. The exclusion criteria were as follows: (1) radiation therapy before NACT; (2) incomplete MR sequence or clinicopathological information; and (3) complications with other malignant tumors. Ultimately, a total of 220 patients from center 1 and 65 patients from center 2 were enrolled according to the selection criteria. The overall radiomics pipeline of this study is shown in Figure 2.

Regular follow-up was performed every 3–6 months for the first 2 years following treatment, every 6 months for the next 3–5 years and then once a year after that. The period from the date of surgery to the date of the first locoregional recurrence, distant metastasis, death, or the last visit in follow-up, which was defined as DFS, was the endpoint of our study. Gynecological examination and imaging modalities such as CT, MRI and positron emission tomography-computed tomography were applied to screen for locoregional recurrence and distant metastases.

### 2.2. Assessment of the NACT Response

All participants conducted one or two cycles of NACT and hysterectomies within 2 weeks. The short-term response was measured by the change in tumor size, which was calculated using the Response Evaluation Criteria In Solid Tumors (RECIST v. 1.1) [31]. Before surgery, the clinical response was assessed and categorized as follows: patients who had complete response (CR) or partial response (PR) were assigned to the pathological good responder (pGR) group, while those who had stable disease (SD) or progressive disease (PD) were assigned to the non-pGR group.

### 2.3. MRI Acquisition

All patients received a systematic pelvic MR examination with a 3.0 T MR scanner at two distinct time periods. The pre- and post-treatment scanning periods were set to be within one week of the initiation of NACT and one week after the completion of NACT, respectively. The MRI parameters of the axial T2WI, DWI and CE-T1WI sequences are detailed in Table S1.

### 2.4. Image Segmentation and Processing

A radiologist with 9 years of experience in abdominal MRI diagnosis delineated regions of interest (ROIs) for use in radiomics analysis slice-by-slice along the tumor boundary manually by ITK-SNAP software (version 3.8.0, open-source software available for download at http://www.itksnap.org, accessed on 1 March 2022). All segmentations underwent quality control for proper annotation by an expert pelvic radiologist (with 15 years of experience in abdominal MRI diagnosis). After three months, 40 patients in the training set were selected randomly and resegmented by the radiologists to confirm the stability of radiomics characteristics.

For DL feature extraction, MR images were cropped by defining a rectangular ROI that encompassed the labeled tumor. Then, to satisfy the input size requirement of the pretrained convolutional neural network (CNN) model, the tumor patch was adjusted to a 224 × 224 square, and the intensity of the tumor patch images was standardized to the color range of 0–255 for training. To improve feature discrimination, many preprocessing procedures were applied before extracting quantitative features. Nonlinear intensity normalization and gray-level quantization were used to transform MR images to standardized intensity ranges and map the whole intensity range of the tumor location to distinct gray levels [32,33]. Finally, images were resampled to an isotropic pixel size using bilinear interpolation. Supplementary S2 contains detailed information about image processing.

### 2.5. Feature Extraction

Two types of features, handcrafted and DL-based radiomics features, were extracted. The open-source Python (v.3.10; https://www.python.org/, accessed on 1 May 2022) toolbox PyRadiomics was used for all handcrafted radiomics features. In total, 1223 handcrafted radiomics features were extracted from each MR sequence, including shape, first-order statistical, texture and transformation features. The details of the feature extraction are shown in Supplementary S3. In this study, DL-based features were extracted using DenseNet-121, which was pretrained on the natural picture dataset ImageNet. All convolutional layers were produced with a constant kernel size of 3 × 3 with stride and padding set to 1. The network consists of 5 max-pooling layers with a window size of 2 × 2 and a nonlinear activation function of rectified linear units, with the final fully connected layer having 1001 features with SoftMax enabled.

### 2.6. Feature Selection and Radiomics Signature Construction

Handcrafted radiomics and DL-based features were normalized individually using Z scores to achieve a standard normal distribution of feature intensity. The following steps were conducted in the training dataset for feature selection and signature establishment. First, the intraclass correlation coefficient (ICC) was determined to confirm the stability of radiomics characteristics, and features with an ICC < 0.85 were removed. Second, univariate analysis was applied to select handcrafted and DL-based radiomics features that were substantially different between the pGR and non-pGR groups using the Mann–Whitney U test. Third, considering that unbalanced classification (pGR group to non-pGR group: 113:35 = 3.23) may lead to inaccurate results, the synthetic minority oversampling technique (SMOTE) was used to balance the minority class in the training dataset so that the ratio of the two groups of patients was set to 1.10:1 (118 patients in the pGR group and 107 in the non-pGR group) in the SMOTE training dataset [34]. Further details of the SMOTE process are shown in Supplementary S4. The SMOTE algorithm was applied to create synthetic samples according to k-nearest neighbour of each minority class sample to balance the number of two-class sample [35,36]. Fourth, to perform feature selection, least absolute shrinkage and selection operator (LASSO) regression with 10-fold cross-validation was utilized. The handcrafted and DL-based radiomics features derived by feature selection were passed through a support vector machine (SVM) classifier to develop the handcrafted radiomics signature and the DL-based radiomics signature, respectively, for predicting the response to NACT.

### 2.7. Clinical Model and DLRN Development and Validation

To investigate the relationship between clinical variables and NACT response, differences in clinical characteristics were evaluated using the chi-square test or Fisher’s exact test for categorical variables and the t test or Mann–Whitney U test for continuous data as applicable. The significant clinical characteristics were then subjected to stepwise backward multivariate logistic regression to determine the independent predictors of the NACT response. The criterion for variable inclusion was *p* < 0.1 for main effects and *p* < 0.05 for interactions. The clinical model comprising only the significant clinical information was constructed.

In this study, as a hybrid model, the DLRN was developed to investigate the potential combined utility of the clinical model, handcrafted radiomics signature, and DL-based radiomics signature by multivariate logistic regression. Given the considerable interpretability of logistic regression, the effect of different models on final prediction outcomes based on model weights may be explained. It is also appropriate for binary classification applications and effective training. The DLRN not only predicts whether the patient would respond to NACT but also gives an approximate probability prediction.

### 2.8. Statistical Analysis

The receiver operating characteristic (ROC) curve was drawn to assess the performances of the handcrafted and DL-based radiomics signatures, clinical model and the DLRN in training and validation cohorts. DeLong’s test was used to compare the AUCs estimation between these prediction models. Performance measurements included sensitivity, specificity and accuracy. Calibration curves and the Hosmer–Lemeshow (H-L) test, which investigate how similar the models’ predictions are to the observed outcome, were implemented to verify calibration performance. To assess the clinical value of models, a decision curve analysis (DCA) was performed by quantifying the net benefits to a range of threshold probabilities in both internal and external validation datasets. To investigate the association between the DLRN score and DFS, the Kaplan–Meier curve was employed, and patients were classified into high-risk or low-risk groups. The DFS prediction model was designed using both univariate and multivariate analyses, as well as Cox proportional hazards regression. The scaled Schoenfeld residual test was used to validate the models’ proportional hazards assumption. Statistical analyses were conducted using R studio software (R Studio Inc., version 4.0.0, R Foundation for Statistical Computing, Vienna, Austria) and SPSS 24.0 software (SPSS Inc., Chicago, IL, USA). The R software packages are provided in the Supplementary S5. A two-sided *p* < 0.05 was considered statistically significant.

## 3. Results

### 3.1. Patient Characteristics

From January 2015 to June 2021, 220 patients from center 1 were included and split into training (*n* = 148; mean age, 52.3 ± 9.54) and validation datasets (*n* = 72; mean age, 51.2 ± 10.52). In addition, 65 patients (mean age, 53.3 ± 10.72) were enrolled from another center as an external validation dataset. The NACT response rate was consistent throughout the three groups of patients, with 76.35% for the training and 63.89% and 72.31% for the internal and external validation datasets, respectively. The baseline clinical characteristics are summarized in Table 1. No significant difference was observed in menopausal status, maximum tumor diameter, pathological type, neutrophil-to-lymphocyte ratio (NLR) or platelet-to-lymphocyte ratio (PLR) between the pGR and non-pGR groups in the datasets (*p* > 0.05). Moreover, age, FIGO stage, serum SCC-Ag (squamous cell carcinoma antigen) level, lymphovascular space invasion (LVSI) status, lymph node metastasis and parametrial invasion (PMI) showed significant differences between the two groups in the training dataset (*p* < 0.05).

### 3.2. Feature Selection and Radiomics Signature Development

**Figure 3:**
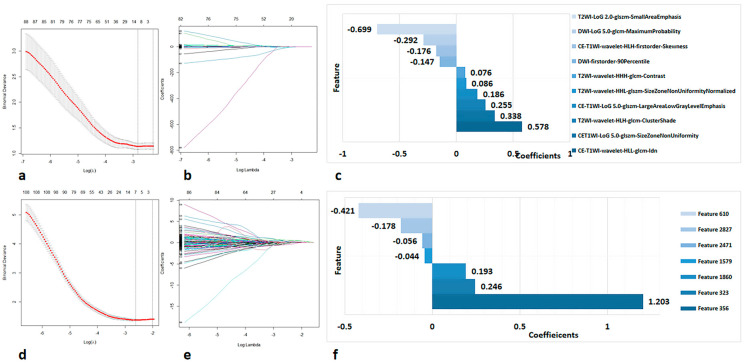
The lasso plots for radiomics feature selection: (**a**,**b**) for handcrafted radiomics features; (**c**) 10 features and corresponding coefficients; (**d**,**e**) for DL-based radiomics features; (**f**) 7 features and corresponding coefficients.

In this study, two types of radiomics features were extracted, including handcrafted radiomics and DL-based radiomics features. For each patient, 3669 handcrafted radiomics features were derived. By univariate analysis, 989 handcrafted radiomics features were found to be significantly different between patients in the pGR and non-pGR groups in the training dataset. As shown in Figure 3a–c, the LASSO classifier selected 10 features (presented in Table 2), including four T2WI features, two DWI features and four CE-T1WI features.

**Figure 4:**
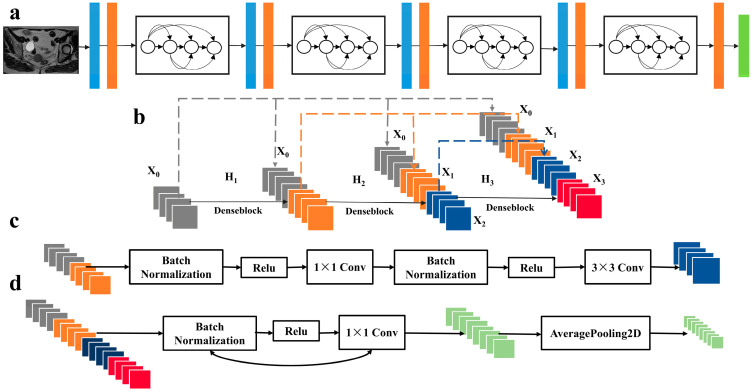
The framework of Densenet-121 model for deep learning-based radiomics feature extraction (**a**). The structures of Dense block (**b**), convolutional block (**c**), and transition layer (**d**) are shown separately.

A total of 3000 DL-based features were extracted using DenseNet-121 (Figure 4 and Supplementary Table S2), and 7 optimal DL-based features were acquired through feature selection, including three T2WI features, two DWI features, and two CE-T1WI features (Figure 3d,e and Table 2). Then, we developed a DL-based radiomics signature to differentiate responders from nonresponders in the training data.

### 3.3. Performance of Handcrafted Radiomics Signatures

The handcrafted radiomics signature achieved an AUC of 0.884 (95% CI, 0.827–0.942) in the training set and 0.858 (95% CI, 0.763–0.953) and 0.810 (95% CI, 0.707–0.912) in the internal and external validation datasets, respectively. The resulting radiomics signature performed stably in all three datasets, and the inaccuracy was within acceptable limits. The internal validation dataset had an accuracy, sensitivity and specificity of 0.781, 0.846 and 0.619, respectively, whereas the corresponding values in the external validation dataset were 0.764, 0.824 and 0.619, respectively.

### 3.4. Performance of the DL-Based Radiomics Signatures

The DL-based radiomics signature yielded AUCs of 0.871 (95% CI 0.756–0.901) in the training dataset, and 0.893 (95% CI 0.814–0.972) and 0.829 (95% CI 0.680–0.936) in these two validation datasets, respectively. The prediction performance of the DL-based signature was slightly higher than that of the handcrafted radiomics signature in the validation dataset.

### 3.5. Performance of the Clinical Model

FIGO stage, serum SCC-Ag level, LVSI status and PMI were identified as independent predictors based on univariate and multivariate logistic regression analyses (*p* < 0.05) in the training dataset and were used to build the clinical model (Table 3). The prediction efficiency was determined for the clinical model in the training dataset (AUC = 0.711; 95% CI: 0.620–0.801), internal validation dataset (AUC = 0.665; 95% CI: 0.538–0.792) and external validation dataset (AUC = 0.689; 95% CI 0.750–0.901).

### 3.6. Performance of the DLRN and Model Comparison

**Figure 5:**
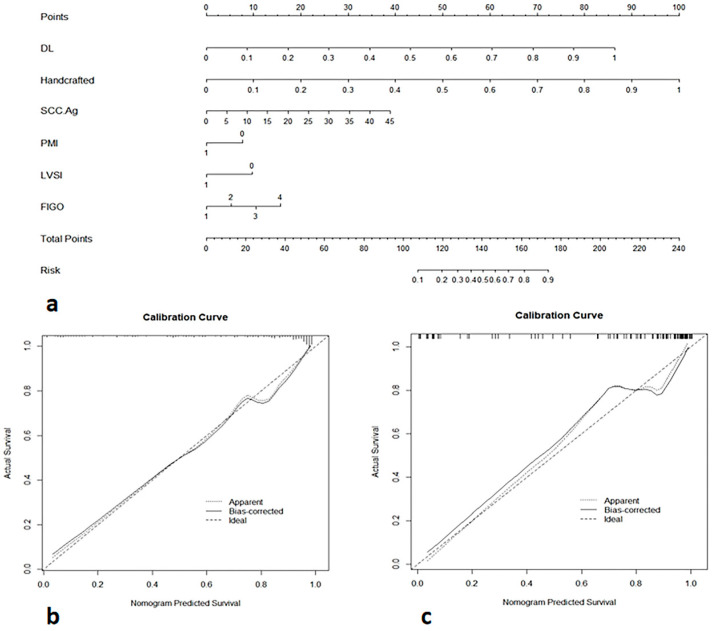
Deep learning radiomics nomogram (DLRN) developed for prediction of response to NACT. (**a**) DLRN combined with the handcrafted and DL-based radiomics signatures and clinical model. Calibration curve of the nomogram in the internal validation (**b**) dataset and external validation (**c**) dataset.

**Figure 6:**
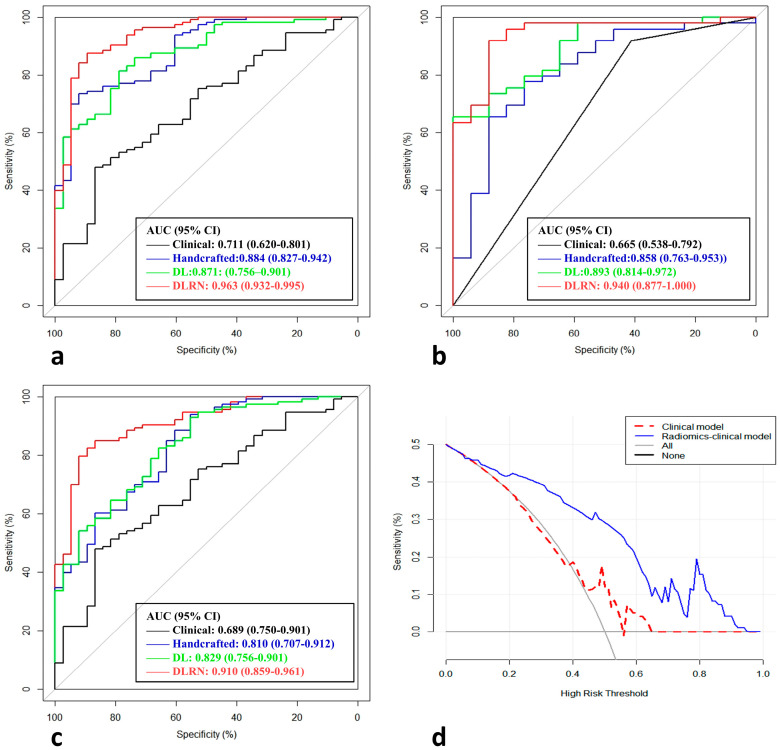
Performance of handcrafted and DL-based radiomics signatures, clinical model and the DLRN. Receiver operating characteristic (ROC) curves of the four models in the training (**a**) dataset, internal validation (**b**) dataset and external validation (**c**) dataset and (**d**) decision curve analysis.

**Figure 7:**
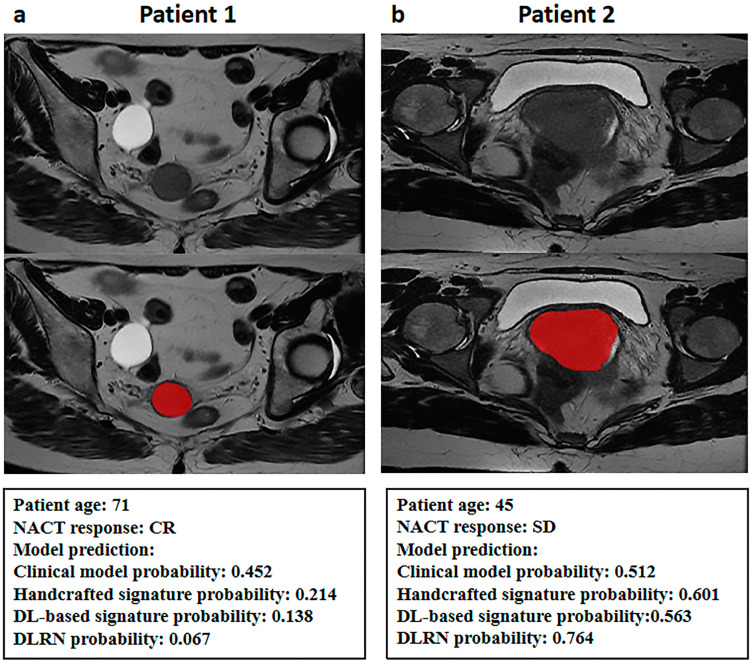
Two typical cases with MR images, clinical characteristics, and predicted results of response to NACT by different models. The red area represented the tumor issue. A 71-year-old woman (**a**) with complete response (CR). The probability of chemoresistance predicted by clinical model, handcrafted radiomics signature and DL-based signature was 0.452, 0.214 and 0.138, and that of chemoresistance predicted by the DLRN was 0.067. A 45-year-old woman (**b**) with stable disease (SD). The probability of chemoresistance predicted by clinical model, handcrafted radiomics signature and DL-based signature was 0.512, 0.601 and 0.5133, and that of chemoresistance predicted by the DLRN was 0.764. T2WI = T2-weighted imaging; DWI = diffusion-weighted imaging; CE-T1WI = contrast-enhanced-T1 weighted imaging.

The handcrafted and DL-based radiomics signatures and the abovementioned independent predictors were incorporated to build the DLRN (Figure 5a and Table 4), which showed good performance for NACT response prediction, achieving AUCs of 0.963 (95% CI 0.932–0.995), 0.940 (95% CI 0.877–1.000) and 0.910 (95% CI: 0.859–0.961) in the training, internal validation and external validation datasets, respectively (Table 5 and Figure 6). The calibration curve showed good agreement between the DLRN predicted response and the actual response to NACT (Figure 5b,c), which was also demonstrated by the Hosmer–Lemeshow test. Compared with the clinical model and the handcrafted and DL-based radiomics signatures, the DLRN provided the best performance in predicting the response to NACT (*p* < 0.05) and was externally validated. The patients who had CR and SD to NACT are shown in Figure 7. Compared with the clinical model, the handcrafted radiomics signature and the DL-based signature, the prediction efficiency of DLRN is more accurate, by considering the three models above. This suggests that the DLRN built in this study was more generalizable and allows for more personalized clinical decision making.

**Figure 8:**
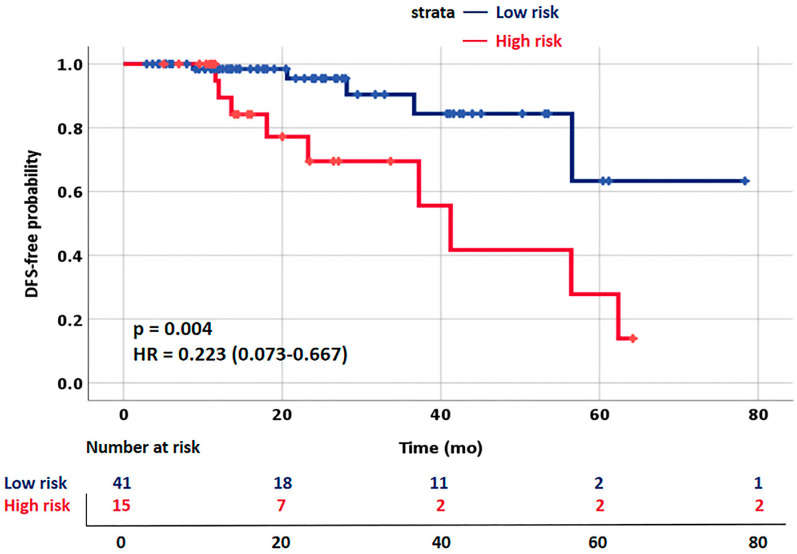
Kaplan–Meier curves of disease-free survival (DFS) on the follow-up LACC dataset.

Furthermore, we looked into the prognosis of DLRN in 101 patients with LACC. The median duration of the follow-up was 29 (range, 16–40) months. As shown in Figure 8, higher DLRN scores were demonstrated to be substantially related to improved DFS (hazard ratio (HR), 0.223; 95% CI, 0.073–0.667, log-rank test, *p* = 0.004).

## 4. Discussion

In this study, two sets of noninvasive radiomics signatures were developed by handcrafted and DL-based radiomics features based on multiparametric MRI to preoperatively predict the clinical response to NACT in patients with LACC. Furthermore, we incorporated these radiomics signatures with clinical independent predictors, namely, serum SCC-Ag level, PMI, LVSI status and FIGO stage, to build a DL radiomics model, which was presented in the form of a nomogram (DLRN). To the best of our knowledge, this is the first study to explore the predictive value of three ensemble models: clinical model, handcrafted radiomics signature and DL-based radiomics signature. The nomogram might be utilized for multidisciplinary consultation between radiologists and oncologists to predict the NACT response preoperatively and propose appropriate tailored therapy alternatives to these patients, rather than depending exclusively on clinical assessment. Furthermore, the DLRN score was substantially correlated with DFS in patients with LACC, yielding essential supplemental information for prognosis.

Predicting the response to NACT before treatment would be extremely beneficial in terms of preventing unnecessary chemotherapy before surgery, as well as for providing treatment options, individualization and effective patient stratification. An increasing number of methods have been shown to predict the response to NACT in patients with LACC. Tian et al. [15] found that CT-based machine learning analysis could be employed to identify whether NACT is effective for patients with LACC, and their hybrid model, which included radiomics features and clinical features, achieved discriminatory performance with AUCs of 0.803 and 0.821 in the training and validation datasets, respectively. However, when compared to CT, MR is a more effective technique for pelvic examinations in terms of tracking disease progression and the effectiveness of treatment [37,38]. Sun et al. [14] showed that a model based on MRI data could yield excellent accuracy in determining the response to NACT by radiomics analysis. Their model achieved a high AUC (0.999 [95% CI 0.997–1]) and specificity (100% [95% CI 99–100%]) in the validation cohort. The results were higher than the result of the handcrafted radiomics signature in our study. The multicenter statistics in their analysis improved the model’s stability, which is worth exploring in future studies.

Previous studies on quantitative image-based prediction have shown that DL-based features, featuring in-depth information in the hidden layers of neural networks without the need for specified features, are complementary to radiomics features [39,40]. Our study demonstrates that this remains true when using DL-based features extracted by Resnet-121, with AUCs that were slightly higher than those of the handcrafted radiomics signature in terms of discrimination for all three datasets. These features have the potential to improve diagnostic value beyond the basic quantification of information currently available in MR images, since they are not being limited to previously established image properties [41,42]. DL-based features provide abundant information on tumor geographic heterogeneity and the tumor microenvironment, both of which are linked to tumor chemical sensitivity, and have utility for cervical cancer screening, detection and prognosis [43,44].

In our study, we extracted radiomics features from MRI, identified the optimal radiomics features, and created a radiomics signature. Likewise, we extracted deep semantic features that can well characterize the tumor tissue to build a DL signature. Finally, the DLRN was developed by incorporating both radiomics signatures with clinical predictors, and its effectiveness was considerably greater than that of the clinical model in all datasets. Earlier studies have found association between FIGO stage and serum SCC-Ag level and NACT effectiveness [29,45]. Our findings are in line with those of Ou et al. [29], who showed that PMI may have an effect on NACT efficacy in LACC. The number of invasive tumor cells is higher in cervical cancer with PMI, which is frequently exposed to strong genetic stress under hypoxic conditions, leading to genetic instability and drug-resistance induction via various mechanisms. Additionally, since cancer stem cells coexist with invasive tumor cells, invasion into surrounding tissue is a significant contribution to therapy response [46,47]. Similarly, the DLRN outperformed the handcrafted and DL-based radiomics signatures in prediction ability in most datasets. Solely DL classification algorithms are prone to overfitting, which may lead to instability; therefore, well-defined handcrafted features were introduced into our processing. The combination of handcrafted and DL-based radiomics signature has the potential to provide higher investment possibilities.

Intriguingly, the score of the DLRN created in this study was strongly related to the DFS of LACC patients. Several studies shown that the response to NACT is a predictor of prognosis and that patients who achieve an NACT response have a better prognosis. Individuals at low risk of an NACT response had a substantially lower chance of favorable long-term results than those at high risk, even after NACT followed by surgery. Patients with higher DLRN scores had a considerably improved DFS. Patients with low DLRN scores tended to have unsatisfactory outcomes; therefore, other treatment options should be provided in a timely manner to minimize excessive toxicity and improve patients’ survival prospects. The high-precision online DLRN will not only aid in the evaluation of treatment options and administration of individualized treatment but also enable clinicians to examine patients remotely.

There are some limitations in our study. First, the number of patients enrolled in this research was still limited, and the patients were all Chinese. Therefore, while data used for modeling and validation were obtained from two centers, additional large-scale worldwide validation is required before the model may be used in patients of other ethnicities. Second, only LACC patients with NACT followed by radical hysterectomy were included; those with NACT followed by radiotherapy were excluded, and all those patients will be enrolled for further analysis for predicting the NACT response. Third, image segmentation was performed manually by radiologists in this study, which was time consuming and laborious. An automatic segmentation model of cervical cancer based on multiple MR sequences should be established. Finally, information included in pathological images and genomes may be valuable for prognostic prediction tasks, implying that larger well-designed prospective studies with numerous data points should be conducted in the future.

## 5. Conclusions

In summary, we developed and validated an MRI-based radiomics nomogram that integrated handcrafted and DL-based radiomics signatures and a clinical model for predicting the response to NACT for patients with LACC preoperatively, which has the potential to become an automatic tool for enabling individualized treatment. Additional validation is needed to generate stronger evidence. The proposed DLRN, incorporating MR-based radiomics signatures and clinical predictors, exhibited promising performance for predicting response and clinical outcomes, and provided valuable information for individualized treatment in LACC. Nevertheless, future prospective investigations with multicenter and large sample sizes are needed to prove the clinical applicability of this DLRN, and radiomics feature explanation at a biological level should be performed in future radiogenomics research.

## Figures and Tables

**Table 1:** Clinicopathological characteristics of patients in the training and validation datasets.

Characteristics	Training Dataset (148)		*p*	Internal Validation Dataset (72)		*p*	External Validation Dataset (65)		*p*
	pGR	Non-pGR		pGR	Non-pGR		pGR	Non-pGR	
Number	113	35		46	26		47	18	
Age, mean ± SD, years	53.7 ± 10.64	51.9 ± 11.85	0.003	51.6 ± 10.21	50.7 ± 9.65	0.009	53.9 ± 11.26	51.3 ± 10.93	0.002
Menopausal status									
No	73 (64.60%)	19 (54.29%)		34 (73.91%)	18 (69.23%)		36 (76.60%)	10 (55.56%)	
Yes	40 (35.40%)	16 (45.71%)	0.272	12 (26.09%)	8 (30.77%)	0.670	11 (23.40%)	8 (44.44%)	0.095
FIGO stage									
IB2	23 (20.35%)	8 (22.86%)		8 (17.39%)	5 (19.23%)		12 (25.53%)	6 (33.33%)	
IIA-IIA2	61 (53.98%)	11 (31.43%)		21 (45.65%)	6 (23.08%)		21 (44.68%)	5 (27.78%)	
IIB-IIIB	29 (25.67%)	16 (45.71%)	0.041	17 (36.96%)	15 (57.69%)	0.143	14 (29.79%)	7 (38.89%)	0.461
Maximum tumor diameter	4.46 ± 1.52	5.02 ± 1.24	0.413	4.92 ± 1.39	4.84 ± 1.28	0.934	4.37 ± 1.72	5.29 ± 1.91	0.845
Pathologic type									
Squamous cell carcinoma	93 (82.30%)	24 (68.57%)		36 (78.26%)	23 (88.46%)		34 (72.34%)	15 (83.33%)	
Adenocarcinoma	20 (17.70%)	11 (31.43%)	0.081	9 (19.57%)	3 (11.54%)	0.557	13 (27.66%)	3 (16.67%)	0.549
SCC-Ag	5.20 (1.90, 9.90)	3.40 (0.80, 10.50)	<0.001	4.45 (1.25, 13.50)	3.65 (0.95, 6.65)	<0.001	5.00 (0.85, 13.65)	3.65 (1.15, 11.55)	<0.001
NLR	4.57 ± 2.94	3.23 ± 2.01	<0.001	4.92 ± 1.96	3.01 ± 2.08	<0.001	3.98 ± 2.29	2.94 ± 2.66	<0.001
PLR	214.83 ± 61.93	201.32 ± 72.93	0.119	193.83 ± 73.84	210.83 ± 73.93	0.081	209.14 ± 64.39	219.67 ± 62.92	0.063
LVSI									
Positive	31 (27.43%)	24 (68.57%)		16 (34.78%)	19 (73.08%)		13 (27.66%)	10 (55.56%)	
Negative	82 (72.57%)	11 (31.43%)	<0.001	30 (65.22%)	7 (26.92%)	0.002	34 (72.34%)	8 (44.44%)	0.035
LNM									
Positive	15 (13.27%)	11 (31.43%)		8 (17.39%)	6 (23.08%)		9 (19.15%)	5 (27.78%)	
Negative	98 (86.73%)	24 (68.57%)	0.014	38 (82.61%)	20 (76.92%)	0.558	38 (80.85%)	13 (72.22%)	0.449
PMI									
Positive	29 (25.66%)	19 (54.29%)		14 (30.43%)	15 (57.69%)		19 (40.43%)	10 (55.56%)	
Negative	84 (74.34%)	16 (45.71%)	0.002	32 (69.57%)	11 (42.31%)	0.024	28 (59.57%)	8 (44.44%)	0.272

pGR = pathological good responder; FIGO = International Federation of Gynecology and Obstetrics; SCC-Ag = squamous cell carcinoma antigen; NLR = neutrophil-to-lymphocyte ratio; PLR = platelet-to-lymphocyte ratio; LVSI = lymphovascular space invasion; LNM = lymph node metastasis; PMI = parametrial invasion.

**Table 2:** The selected handcrafted and DL-based radiomics features and coefficients for radiomics signatures.

Handcrafted radiomics features	**Sequence**	**Selected Features**	**Coefficients**
	T2WI	LoG 2.0_glszm_SmallAreaEmphasis	0.578
	T2WI	Wavelet-HHL_glszm_SizeZoneNonUniformityNormalized	0.076
	T2WI	Wavelet-HHH_glcm_Contrast	0.086
	T2WI	Wavelet-HLH_glcm_ClusterShade	−0.176
	DWI	Firstorder_90Percentile	0.186
	DWI	LoG 5.0_glcm_MaximumProbability	0.338
	CE-T1WI	LoG 5.0_glszm_LargeAreaLowGrayLevelEmphasis	−0.147
	CE-T1WI	Wavelet-HLH_firstorder_Skewness	0.255
	CET1WI	LoG 5.0_glszm_SizeZoneNonUniformity	−0.292
	CE-T1WI	Wavelet-HLL_glcm_Idn	−0.699
DL-based radiomics features	T2WI	Feature 323	−0.178
	T2WI	Feature 356	−0.421
	T2WI	Feature 610	1.203
	DWI	Feature 1579	−0.044
	DWI	Feature 1860	−0.056
	CE-T1WI	Feature 2471	0.193
	CE-T1WI	Feature 2827	0.246

**Table 3:** Results of the univariate and multivariate logistic regression analyses based on the training cohort.

Variables	Univariate Logistic Regression Analysis		Multivariate Logistic Regression Analysis	
	OR (95% CI)	*p*	OR (95% CI)	*p*
Age	1.051 (1.009, 1.086)	<0.05 *	-	-
Menopausal status	-	-	-	-
FIGO stage	1.139 (0.796, 1.650)	<0.05 *	0.263 (0.113, 0.611)	<0.05 *
Maximum tumor diameter	-	-	-	-
Pathologic type	-	-	-	-
CA-125	-	-	-	-
Serum SCC-Ag level	1.021 (0.985, 1.058)	<0.05 *	1.018 (0.978, 1.060)	<0.05 *
NLR	-	-	-	-
PLR	-	-	-	-
LVSI	0.211 (0.103, 0.432)	<0.05 *	0.263 (0.113, 0.611)	<0.05 *
LNM	0.973 (0.387, 2.443)	<0.05 *	-	-
PMI	0.357 (0.176, 0.728)	<0.05 *	0.617 (0.257, 1.485)	<0.05 *

NLR = neutrophil-to-lymphocyte ratio; PLR = platelet-to-lymphocyte ratio. * Indicates the *p* < 0.05.

**Table 4:** Multivariate logistic regression of the DLRN.

Index	β	Odds Ratio (95% CI)	Multivariate *p* Value
Clinical model probability	1.297	3.660 (1.109–12.082)	0.033 *
Handcrafted radiomics signature probability	1.087	2.965 (1.796–4.893)	<0.001 *
DL-based radiomics signature probability	1.157	3.181 (1.845–5.486)	<0.001 *
Intercept	−0.547	-	0.078

Combined model probability = sigmoid (1.297 × Clinical model probability + 1.087 × Handcrafted radiomics signature probability + 1.157 × DL-based radiomics signature probability − 0.547). * Indicates the β is the regression coefficient; the *p* < 0.05.

**Table 5:** Predictive performances of clinical model, radiomics model based on mpMRI and radiomics clinical nomogram.

	Training Dataset				Internal Validation Dataset				External Validation Dataset			
	AUC	Acc ^a^	Sen ^a^	Spe ^a^	AUC	Acc ^a^	Sen ^a^	Spec ^a^	AUC	Acc ^a^	Sen ^a^	Spe ^a^
Handcrafted signature	0.884 (0.827–0.942)	0.795	0.858	0.605	0.858 (0.763–0.953)	0.781	0.846	0.619	0.810 (0.707–0.91)	0.764	0.824	0.619
DL-based signature	0.871 (0.756–0.901)	0.821	0.859	0.711	0.893 (0.814–0.972)	0.833	0.898	0.710	0.829 (0.756–0.90)	0.788	0.867	0.625
Clinical model	0.711 (0.620–0.801)	0.730	0.877	0.389	0.665 (0.538–0.792)	0.788	0.918	0.412	0.689 (0.750–0.90)	0.728	0.885	0.363
DLRN	0.963 (0.932–0.995)	0.927	0.946	0.868	0.940 (0.877–1.000)	0.909	0.918	0.882	0.910 (0.859–0.96)	0.854	0.903	0.830
Handcrafted vs. DL signature	0.376				0.238				0.303			
Handcrafted vs. Clinical model	<0.001				0.014				0.022			
DLRN vs. Handcrafted	<0.001				0.042				0.047			
DLRN vs. Clinical model	<0.001				<0.001				<0.001			
DLRN vs. DL signature	0.003				0.251				0.016			

The DLRN indicates deep learning radiomics nomogram, which integrated handcrafted signature, DL-based signature and clinical model; ^a^ Sen, Spe, Acc and AUC indicate the sensitivity, specificity, accuracy and area under the curve of the receiver operating characteristic curve, respectively.

## Data Availability

The data presented in this study are available on request from the corresponding author. The data are not publicly available due to privacy restrictions.

## Supplementary Materials

The following supporting information can be downloaded at: https://www.mdpi.com/article/10.3390/diagnostics13061073/s1.
