# Supplementary material for: A Deep Learning Radiomics Nomogram to Predict Response to Neoadjuvant Chemotherapy for Locally Advanced Cervical Cancer: A Two-Center Study
Source: Diagnostics (Basel). 2023 Mar 11;13(6):1073. doi: 10.3390/diagnostics13061073 (PMC10047639; doi:10.3390/diagnostics13061073)
Supplement: Supplementary file 1 [file diagnostics-13-01073-s001.zip › diagnostics-2253660-supplementary.pdf]

Supplementary Material

Title: A deep learning radiomics nomogram to predict response to neoadjuvant chemotherapy for locally advanced cervical cancer: A two-center study

Supplementary S1: The Sample Size estimation

The sample size of the training dataset and internal and external validation datasets were assessed. To avoid overfitting, the number of predictors should be kept within 1/20-1/8 of the sample size in the training dataset. In this study, there are 142 patients in the training dataset. 10 handcrafted and 8 DL-based radiomcs features were selected to build deep learning radiomics nomogram (DLRN) to predict NACT response.

Supplementary S2: Tumor Segmentation and Image Preprocessing

All patients enrolled in both centers had scan settings that were similar but with distinct systems and parameters. To improve feature discrimination, many preprocessing procedures were applied before extracting quantitative features. Nonlinear intensity normalization and gray-level quantization were used to transform MR images to standardized intensity ranges and map the whole intensity range of the tumor location to distinct gray levels [1, 2]. Finally, images were resampled to an isotropic pixel size using bilinear interpolation.

For deep features, we cropped the MR images by finding a rectangular ROI that enclosed the outlined tumor. Then we resized the tumor patch to a 224 × 224 square to fulfill the requirement for the input size of the pretrained CNN model that we used. Also considering that the CNN model that we used was pretrained on natural images with a color range of 0－255, we normalized the intensity of tumor patch images to the same color range. By determining a rectangular ROI that contained the tumor contour, we cropped the MR images in order to extract their deep features. The tumor patch was then adjusted to a 224 × 224 square to meet the input size requirement of the pretrained CNN model that we utilized. Given that the CNN model we utilized was trained on natural pictures with a color range of 0-255, the intensity of tumor patch images was normalized to the same color range.

[1] Collewet, G.; Strzelecki, M.; Mariette, F. Influence of MRI acquisition protocols and image intensity normalization methods on texture classification. Magn. Reson. Imaging. 2004, 22, 81–91.

[2] Gibbs, P.; Turnbull, L.-W. Textural analysis of contrast-enhanced MR images of the breast. Magn. Reson. Med. 2003, 50, 92–98.

Supplementary S3: Feature extraction methodology

Feature extraction included handcrafted radiomics features and deep learning based radiomics features. Note that, all features were normalized by Z-score method into a standardized value range.

Axial T2WI, DWI and CE-T1WI images retrieved from the picture archiving and communication system (PACS, Carestream), which were then loaded into ITK-SNAP software (3.8.0, www.itksnap.org) for manual segmentation. The segmentation files were stored in Neuroimaging Informatics Technology Initiative (NIfTI) format. Radiologists were blinded to the patients' clinical information. Since the boundary between a tumor and nearby normal soft tissues is not always well defined, a conservative approach to contouring was taken to remain within the tumor, even at the risk of not including a small part of tumor edges. As a result, such VOI was defined for each patient for radiomics feature extraction.

Details of the pyradiomics code is as follow:

```
import numpy as np
import pandas as pd
from radiomics import featureextractor
from sklearn.utils import shuffle
import os
import seaborn as sns
import SimpleITK as sitk
import radiomics
dataDir = 'address//'
para = 'C://Users//user//Params2.yaml'
extractor = featureextractor.RadiomicsFeatureExtractor(para)
folderList = os.listdir(dataDir)
df = pd.DataFrame()
for folder in folderList:
    imageName = dataDir+folder+'//data.nii'
    maskName = dataDir+folder+'//mask.nii'
    featureVector = extractor.execute(imageName,maskName)
```

```
        df_add = pd.DataFrame.from_dict(featureVector.values()).T
        df_add.columns = featureVector.keys()
        df_add.insert(0,'Patient',folder)
        df = pd.concat([df,df_add])
        print(str(folder)+'extracted feature')
df.to_excel(dataDir+'name.xlsx')
```

In our study, a total of 3669 imaging features for each patient were extracted, of which 1223 features were from the three examined modalities (T2W, DWI and CET1W), respectively. All radiomics features were calculated automatically with the Pyradiomics software. The 1223 features could be divided into these categories:

a. 234 first-order statistics features,

b. 14 shape-based features,

c. 312 gray level co-occurrence matrix (GLCM) features,

d. 208 gray level run length matrix (GLRLM) features,

e. 208 gray level size zone matrix (GLSZM) features,

f. 65 neighboring gray tone difference matrix (NGTDM) features,

g. 182 gray level dependence matrix (GLDM) features.

The detailed information of these features was available in the documentation for PyRadiomics, http://PyRadiomics.readthedocs.io/en/latest/).

(1) First-order statistics features

First-order statistics are generally used and basic metrics to explain the distribution of voxel intensities inside the image region defined by the mask. 234 first-order statistics features were retrieved in total.

(2) Shape-based 3D features

Descriptors of the three-dimensional size and shape of the region of interest were included in this category of characteristics (ROI). Because these characteristics are independent even by gray level intensity distribution in the ROI, they can only be estimated on the non-derived picture and mask. In this work, we retrieved 14 shape-based characteristics.

(3) Statistics-based textural features

Statistics-based textural features can reflect the homogeneity phenomenon of the images and

the arrangement of the properties that change slowly or periodically on the body surface. Textural features extracted in our study included types of matrix features, including 312 gray-level co-occurrence matrix (GLCM) features, 208 gray-level run length matrix (GLRLM) features, 208 gray-level size zone matrix (GLSZM) features, 65 Neighbouring Gray Tone Difference Matrix (NGTDM), and 182 gray-level dependence matrix (GLDM) features. Determining the texture matrix representations requires the voxel intensity values within the volume of interest (VOI) to be discretized. Voxel intensities were therefore resampled into equally spaced bins using a bin-width of 25 gray levels. This discretization step not only reduces the image noise but also normalizes the intensities across all patients, allowing for a direct comparison of all the calculated textural features between patients.

A GLCM defines each pixel's distance and angle and determines the correlation between two gray levels with different directions and distances. GLCM can reflect integrated information about the images' direction, interval, amplitude, and frequency. The run length metrics in GLRLM quantify the gray level runs in a picture. A gray level run is defined as the number of pixels and the number of consecutive pixels with the same gray-level value. A GLSZM specifies the number of homogenous related zones with a specific size and intensity inside the tumor volume, representing tumor heterogeneity on a regional scale.

(4) Wavelet and LoG filtration features

Two image filters, wavelet and LoG were applied to original image respectively and yield a corresponding derived image. A LoG spatial band-pass filter was used to derive image features at different spatial scales by turning the filter parameter with 3.0 and 5.0. Wavelet transformation effectively decouples the textural information by decomposing the original image in low- and high-frequencies. In our present study, a discrete, one-level and undecimated three-dimensional wavelet transformation was applied to each MRI image, which decomposed the original image into 8 decompositions. Consider L and H to be low-pass and high-pass functions, respectively, X to be the decomposing image, and the wavelet decompositions of X to be labeled as XLLL, XLLH, XLHL, XLHH, XHLL, XHLH, XHHL, XHHH. Then, eight new images that are decomposed in three directions (x, y, z) can be obtained. Since the applied wavelet decomposition is undecimated, the size of each decomposition is equal to the original image and each decomposition is shift invariant. Thus,

the original tumor delineation of the tumor volume can be applied directly to the decompositions after wavelet transformation.

Supplementary S4: Synthetic Minority Oversampling Technique (SMOTE)

The SMOTE algorithm was applied to create synthetic samples according to k-nearest neighbour of each minority class sample to balance the number of two-class sample [3, 4]. The advantage of this method was to obtain the synthetic virtual samples that have similar attribution values to the existing samples, thus enhancing the representation of the minority group while retaining the original structure of the samples. The SMOTE algorithm was performed by using R function of "SMOTE" in "DMwR" package. If there were N1 samples for the minority while N2 samples for the majority in the training dataset, the dataset could be balanced by using SMOTE algorithm at the given oversampling ratio a (%) and undersampling ratio b (%) based On K-nearest algorithm at a given K value. Firstly, the minority class samples were augmented based on K-nearest algorithm (K = 5) to result in a new minority class samples: the minority class sample= N1+a*N1/100Secondly, the majority class samples could be undersampled at a ratio of b (%) to result in a new majority sample: the majority class = (b/100)*(a*N1/100) Finally, the SMOTE training dataset included the new minority and majority class samples.

[3] Agazzi, G.M.; Ravanelli, M.; Roca, E.; et al. CT texture analysis for prediction of EGFR mutational status and ALK rearrangement in patients with non-small cell lung cancer. Radiol. Med. 2021, 126, 786-794.

[4] Wu, H.; Wu, C.; Zheng. H.; et al. Radiogenomics of neuroblastoma in pediatric patients: CT-based radiomics signature in predicting MYCN amplification. Eur. Radiol. 2021, 31, 3080-3089.

Supplementary S5: The Statistical Analysis and Packages of R Software

The ROC curves were plotted using the "pROC" package. Kaplan-Meier curve, nomogram construction and calibration plot were analyzed using the "rms" and "survival" packages. The Hosmer-Lemeshow test was performed using the " Resource Selection" package. Decision curve analysis was performed with the function of "dca.R". All statistical tests were two-sided, and p values of <0.05 were considered significant.

Table S1 All patients underwent standard pretreatment pelvic MRI with 3.0T MR scanners from two centers.

	Center 1						Center 2		
Parameters	GE 3.0T (Signa HDxt)			SIEMENS 3.0T (Magnetom Skyra)			GE 3.0T (Signa HDxt)		
Sequence	T2WI	DWI	CE-T1WI	T2WI	DWI	CE-T1WI	T2WI	DWI	CE-T1WI
TR/TE (ms)	3400/100	7500/50	3.3/1.3	3400/80	4500/60	3.25/1.22	3740/102	4160/30	4.3/1.7
Matrix	288×256	132×128	128×128	256×256	128×128	256×256	320×240	128×128	256×320
Slice gap (mm)	2	0	0	6.1	0	0	1.2	0	0
Slice thickness (mm)	6	3	2	5	4	3	5	3	1

CE-T1WI was obtained using T1-weighted fat-suppression images and a three dimensional (3D) liver acceleration volume acquisition (LAVA) sequence during the injection of 0.2 mmol/kg of magnevist (Gadopentetic Acid Dimeglumine Salt Injection) at a rate of 2.0 ml/s and following a 40-mL saline flush at the same rate. The contrast medium was injected after the acquisition of three sets of pre-contrast T1 mapping.

Table S2 Parameters of the Densenet-121 model

Layers	Output size	Size and stride
**Convolution**	**112×112**	$7\times7\ conv, stride2$
**Pooling**	**56×56**	$3\times3\ \max\ pool, stride2$
**Denseblock (1)**	**56×56**	$\begin{bmatrix}1\times1\ conv\\3\times3\ conv\end{bmatrix}\times6$
**Translation Layer (1)**	**56×56**	$1\times1\ conv$
	**28×28**	$2\times2\ average\ pool, stride2$
**Denseblock (2)**	**28×28**	$\begin{bmatrix}1\times1\ conv\\3\times3\ conv\end{bmatrix}\times12$
**Translation Layer (2)**	**28×28**	$1\times1\ conv$
	**14×14**	$2\times2\ average\ pool, stride2$
**Denseblock (3)**	**14×14**	$\begin{bmatrix}1\times1\ conv\\3\times3\ conv\end{bmatrix}\times24$
**Translation Layer (3)**	**14×14**	$1\times1\ conv$
	**7×7**	$2\times2\ average\ pool, stride2$
**Denseblock (4)**	**7×7**	$\begin{bmatrix}1\times1\ conv\\3\times3\ conv\end{bmatrix}\times16$
**Classification Layer**	**1×1**	$7\times7\ global\ average\ pool$
	**28×28**	$1000D\ fully-connected, soft\max$
